# Antimicrobial Resistance: An Emerging Global Threat to Modern Medicine

**DOI:** 10.7759/cureus.97668

**Published:** 2025-11-24

**Authors:** Esteban Zavaleta-Monestel, Sebastián Arguedas-Chacón, Carolina Rojas-Chinchilla, José Pablo Díaz-Madriz

**Affiliations:** 1 Pharmacy, Hospital Clinica Biblica, San Jose, CRI; 2 Pharmacy, Hospital Clínica Bíblica, San Jose, CRI

**Keywords:** antibiotic stewardship, antimicrobial resistance, drug-resistant infections, global health, one health

## Abstract

Antimicrobial resistance (AMR) constitutes a critical challenge in contemporary healthcare. The proliferation of resistant bacteria threatens the safety of surgical procedures, cancer chemotherapy, and critical care worldwide. Increased mortality from drug-resistant infections results from both microbial adaptation and systemic deficiencies in surveillance, stewardship, and innovation. Although global awareness has increased, the development of new antibiotics remains limited, and disparities in access to effective therapies continue. Addressing this crisis requires a unified One Health approach that integrates human, animal, and environmental health.

This editorial analyzes the scope of AMR, the economic and regulatory factors driving the decline in antibiotic innovation, and the urgent reforms needed to sustain modern medical practice.

## Editorial

Since their introduction in the 1940s, antibiotics have enabled the effective treatment of previously fatal infections and facilitated progress in surgery, transplantation, and intensive care. These advancements are now at considerable risk due to the escalating threat of antimicrobial resistance. The Global Research on Antimicrobial Resistance (2024) estimated that 4.71 million deaths were associated with bacterial antimicrobial resistance (AMR) in 2021, of which 1.14 million were directly attributable to resistant infections [1]. Mortality from AMR now exceeds that of HIV or malaria, demonstrating that AMR constitutes a global health crisis with a burden like other major communicable diseases [2].

The current epidemiological trends are alarming. The World Health Organization’s Global Antimicrobial Resistance and Use Surveillance System (GLASS 2025) reports that approximately one in six bacterial infections globally are resistant to first-line antibiotic therapy [3]. Resistance has increased by over 40% across more than 40 pathogen-drug combinations since 2018, indicating deficiencies in surveillance, infection prevention, and stewardship. Carbapenem-resistant Enterobacterales and *Acinetobacter baumannii* are now prevalent in intensive care units, while community-acquired methicillin-resistant *Staphylococcus aureus* remains widespread despite longstanding control efforts [3,4]. In cases of neonatal and postoperative sepsis, multidrug resistance frequently converts treatable infections into fatal outcomes. Low- and middle-income countries bear a disproportionate burden due to limited diagnostic infrastructure, inadequate antibiotic regulation, and restricted access to reserve drugs. This burden is particularly evident in many African and Asian countries, where at least 60 nations are unlikely to achieve the sustainable development goal target of fewer than 12 neonatal deaths per 1,000 live births by 2030 [5].

The impact of antimicrobial resistance extends beyond infectious diseases. AMR undermines the safety of medical procedures, including organ transplantation, joint replacement, and chemotherapy, all of which depend on effective antibiotics to prevent and treat secondary infections [6]. The loss of effective antimicrobials poses substantial economic risks, potentially undermining decades of productivity and development. The World Bank projects that, if AMR remains uncontrolled, global gross domestic product (GDP) could decline by 3.8% by 2050, with the largest proportional losses in low- and middle-income countries [7]. Beyond healthcare, AMR threatens agriculture, international trade, and food security, representing a systemic risk with both biomedical and macroeconomic consequences [8].

Simultaneously, the pace of new antibiotic development has declined markedly. The World Health Organization's State of the World’s Antibacterials (2024) report identified 97 agents in the clinical and pre-clinical development pipeline, including 57 traditional antibiotics and 40 non-traditional candidates such as bacteriophages and monoclonal antibodies [9]. A few of these agents possess novel mechanisms of action or target critical-priority pathogens. Most represent minor modifications of existing drug classes, leading to incremental rather than transformative therapeutic progress. The research environment is increasingly characterized by diminished innovation and industry consolidation [10].

The reasons are systemic. Antibiotic research is economically highly challenging, with high R&D costs, unpredictable approval pathways, and stewardship policies that necessarily restrict post-market sales [11]. In recent years, several biotechnology companies, including Achaogen, Melinta Therapeutics, and Entasis, publicly filed for bankruptcy soon after launching new antibiotics despite regulatory approval. Their collapse illustrates the paradox of modern antibiotic innovation: the very stewardship measures necessary to preserve efficacy, by limiting prescriptions to truly essential cases, also restrict sales volume and thus revenue, making cost recovery exceedingly difficult. In a pharmaceutical market optimized for chronic and high-volume therapies, the economic model remains structurally incompatible with short-course curative treatments [12,13].

Recent policy measures seek to address insufficient incentives for antibiotic development. The United Kingdom’s National Health Service has adopted a subscription model that provides fixed annual payments for access to two novel antibiotics, decoupling profit from sales volume and ensuring stable revenue for manufacturers [14]. In the United States, the proposed PASTEUR (Pioneering Antimicrobial Subscriptions To End Upsurging Resistance) Act aims to address the antibiotic market failure by offering developers multi-year, fixed payments for critically needed antibiotics, regardless of sales volume. This subscription-based model would decouple revenue from usage, providing financial incentives for the development of new antimicrobials. These strategies represent a shift from traditional volume-based payment models to preparedness-based incentives [15]. However, their effectiveness is constrained by geographic and regulatory limitations. Without coordinated international implementation and harmonized regulatory standards, these incentives are unlikely to attract the global investment required for antibiotic discovery [11].

Efforts to control resistance must extend beyond human medicine. The Quadripartite Alliance of the World Health Organization (WHO), Food and Agriculture Organization (FAO), United Nations Environment Program (UNEP), and World Organization for Animal Health (WOAH) (2024) has established a comprehensive One Health framework that integrates human, animal, and environmental sectors [16]. More than two-thirds of global antibiotic use occurs in livestock and aquaculture, frequently for growth promotion rather than disease treatment. However, usage patterns vary substantially across regions, with particularly intensive and often less-regulated use in parts of Asia, Latin America, and Africa. Resistant organisms and genes from these sectors spread through food, water, and soil, perpetuating a feedback loop among ecosystems [17]. Environmental contamination from pharmaceutical manufacturing further exacerbates the issue. Effective One Health implementation requires integrated surveillance, restrictions on non-therapeutic antibiotic use in agriculture, and investments in sanitation, waste management, and veterinary oversight [18].

In clinical settings, stewardship and diagnostics are the primary defenses against antimicrobial resistance. Hospitals should implement stewardship programs that connect antibiotic prescribing to real-time microbiological data and measurable outcomes. Rapid molecular assays that identify resistance genes within hours can decrease empiric broad-spectrum prescribing and improve patient outcomes; however, these technologies remain expensive and are still primarily available in high-resource settings [19]. Digital dashboards that provide prescribers with real-time resistance data can further improve decision-making. However, equitable access to diagnostics remains limited in low-resource environments, where empirical therapy often replaces laboratory confirmation. Addressing this diagnostic gap is as essential as developing new antibiotics; without accurate data, optimal antibiotic use cannot be achieved [20].

Education is a critical factor in promoting sustainable antimicrobial stewardship. Each antibiotic prescription exerts selective pressure on microbial populations beyond the individual patient [19]. Integrating stewardship principles into medical and nursing curricula, associating institutional accreditation with antibiotic-use metrics, and enhancing public awareness can foster responsible antimicrobial use [21]. Stewardship must be regarded as an ethical responsibility that safeguards public health for future generations. In many countries, particularly in low- and middle-income settings, access to second-line or reserve antibiotics remains scarce or unaffordable, perpetuating the use of ineffective or counterfeit drugs. International financing mechanisms should guarantee access to quality-assured antimicrobials while enforcing global standards for manufacturing and distribution [22]. Ensuring that all countries, regardless of their income level, can access life-saving antibiotics is both a humanitarian duty and a pragmatic necessity. Resistant pathogens do not recognize borders; unchecked inequity anywhere weakens global security everywhere [23].

All proposed interventions must be executed with transparency and ethical rigor, ensuring that financial incentives do not compromise stewardship or patient safety. The perspectives presented are based on global evidence as of 2025 and are intended to inform professional and policy discussions, not to represent official institutional positions [24].

Antimicrobial resistance highlights the interconnectedness of scientific, societal, economic, and biological factors. Surveillance systems provide quantitative data on the scale of the problem, but effective change requires more than measurement [8]. The global response to AMR will determine the trajectory of modern medicine, influencing whether future healthcare is characterized by continued innovation or by increased mortality from previously manageable infections [25]. Preserving the effectiveness of antimicrobials is both a scientific and ethical imperative, requiring coordinated action and sustained political commitment. Immediate, collective efforts are necessary to maintain the therapeutic foundations essential to modern healthcare.

## References

[1] GBD 2021 Antimicrobial Resistance Collaborators (2024). Global burden of bacterial antimicrobial resistance 1990-2021: a systematic analysis with forecasts to 2050. Lancet.

[2] Antimicrobial Resistance Collaborators (2022). Global burden of bacterial antimicrobial resistance in 2019: a systematic analysis. Lancet.

[3] (2025). World Health Organization. Global antibiotic resistance surveillance report 2025: WHO Global Antimicrobial Resistance and Use Surveillance System (GLASS): summary. World Health Organization.

[4] Thacharodi A, Vithlani A, Hassan S, Alqahtani A, Pugazhendhi A (2024). Carbapenem-resistant Acinetobacter baumannii raises global alarm for new antibiotic regimens. iScience.

[5] Dramowski A, Bolton L, Fitzgerald F, Bekker A (2025). Neonatal sepsis in low- and middle-income countries: where are we now?. Pediatr Infect Dis J.

[6] Ahmed SK, Hussein S, Qurbani K, Ibrahim RH, Fareeq A, Mahmood KA, Mohamed MG (2024). Antimicrobial resistance: Impacts, challenges, and future prospects. Journal of Medicine, Surgery, and Public Health.

[7] (2025). World Health Organization. Antimicrobial resistance. https://www.who.int/news-room/fact-sheets/detail/antimicrobial-resistance.

[8] Salam MA, Al-Amin MY, Salam MT, Pawar JS, Akhter N, Rabaan AA, Alqumber MA (2023). Antimicrobial resistance: a growing serious threat for global public health. Healthcare (Basel).

[9] World Health Organization (2024). 2023 Antibacterial agents in clinical and preclinical development: an overview and analysis. 2023 Antibacterial Agents in Clinical and Preclinical Development. An Overview and Analysis.

[10] Niño-Vega GA, Ortiz-Ramírez JA, López-Romero E (2025). Novel antibacterial approaches and therapeutic strategies. Antibiotics (Basel).

[11] Gargate N, Laws M, Rahman KM (2025). Current economic and regulatory challenges in developing antibiotics for Gram-negative bacteria. NPJ Antimicrob Resist.

[12] Mullard A (2019). Achaogen bankruptcy highlights antibacterial development woes. Nat Rev Drug Discov.

[13] Muteeb G, Rehman MT, Shahwan M, Aatif M (2023). Origin of antibiotics and antibiotic resistance, and their impacts on drug development: a narrative review. Pharmaceuticals (Basel).

[14] Barlow E, Morton A, Megiddo I, Colson A (2022). Optimal subscription models to pay for antibiotics. Soc Sci Med.

[15] Spellberg B, Blaser M, Guidos RJ (2011). Combating antimicrobial resistance: policy recommendations to save lives. Clin Infect Dis.

[16] (2025). Quadripartite key recommendations and priorities for the 2024 UNGA high-level meeting on antimicrobial resistance (AMR) - a policy brief. https://www.unep.org/resources/policy-and-strategy/quadripartite-key-recommendations-and-priorities-2024-unga-high-level.

[17] Abate TA, Birhanu AG (2025). Antibiotic use in livestock and environmental antibiotic resistance: a narrative review. Environ Health Insights.

[18] Ohia CM, Falodun OI, Adebudo LI, Bakarey AS (2025). A one health perspective on multidrug-resistant bacterial infections: integrated approaches for surveillance, policy and innovation. Front Cell Infect Microbiol.

[19] Shrestha J, Zahra F, Cannady Cannady, Jr P (2025). Antimicrobial stewardship. StatPearls [Internet].

[20] Garzón-Orjuela N, Parveen S, Amin D, Vornhagen H, Blake C, Vellinga A (2023). The effectiveness of interactive dashboards to optimise antibiotic prescribing in primary care: a systematic review. Antibiotics (Basel).

[21] Balea LB, Gulestø RJ, Xu H, Glasdam S (2024). Physicians', pharmacists', and nurses' education of patients about antibiotic use and antimicrobial resistance in primary care settings: a qualitative systematic literature review. Front Antibiot.

[22] File TM Jr, Srinivasan A, Bartlett JG (2014). Antimicrobial stewardship: importance for patient and public health. Clin Infect Dis.

[23] Ren M, So AD, Chandy SJ (2022). Equitable access to antibiotics: a core element and shared global responsibility for pandemic preparedness and response. J Law Med Ethics.

[24] Jegan R, Rose A, Dierickx K (2025). Institutional financial incentives in healthcare: a review of normative considerations. BMC Med Ethics.

[25] Davies SC, Oxlade C (2021). Innovate to secure the future: the future of modern medicine. Future Healthc J.

